# Voglibose Regulates the Secretion of GLP-1 Accompanied by Amelioration of Ileal Inflammatory Damage and Endoplasmic Reticulum Stress in Diabetic KKAy Mice

**DOI:** 10.3390/ijms232415938

**Published:** 2022-12-14

**Authors:** Yaxin Fu, Wenming Ji, Quan Liu, Lin Zhang, Caina Li, Yi Huan, Lei Lei, Xuefeng Gao, Leilei Chen, Cunyu Feng, Liran Lei, Jiayu Zhai, Pingping Li, Hui Cao, Shuainan Liu, Zhufang Shen

**Affiliations:** 1 State Key Laboratory of Bioactive Substances and Functions of Natural Medicines, Institute of Materia Medica, Chinese Academy of Medical Sciences and Peking Union Medical College, Beijing 100050, China; 2 Key Laboratory of Polymorphic Drugs of Beijing, Institute of Materia Medica, Chinese Academy of Medical Sciences and Peking Union Medical College, Beijing 100050, China; 3 Diabetes Research Center of Chinese Academy of Medical Sciences and Peking Union Medical College, Beijing 100050, China; 4 Department of Endocrinology, Beijing Tongren Hospital, Capital Medical University, Beijing 100730, China; 5 Department of Medical Records, Beijing Tongren Hospital, Capital Medical University, Beijing 100730, China; 6 Beijing Diabetes Institute, Beijing 100730, China

**Keywords:** voglibose, type 2 diabetes, inflammation, ileal macrophages, endoplasmic reticulum stress

## Abstract

Voglibose is an α-glycosidase inhibitor that improves postprandial hyperglycemia and increases glucagon-like peptide-1 (GLP-1) secretion in patients with type 2 diabetes. Recently, there has been increasing interest in the anti-inflammatory effects of voglibose on the intestine, but the underlying mechanism is not clear. This study evaluated the effects and mechanisms of voglibose on glycemic control and intestinal inflammation. Type 2 diabetic KKAy mice were treated with voglibose (1 mg/kg) by oral gavage once daily. After 8 weeks, glucose metabolism, levels of short-chain fatty acids (SCFAs), systematic inflammatory factors, intestinal integrity and inflammation were evaluated using hematoxylin and eosin staining, immunohistochemistry, immunofluorescence and Western blot analysis. Voglibose ameliorated glucose metabolism by enhancing basal- and glucose-dependent GLP-1 secretion. Several beneficial SCFAs, such as acetic acid and propionic acid, were increased by voglibose in the fecal sample. Additionally, voglibose notably decreased the proportion of pro-inflammatory macrophages and the expression of nuclear factor kappa B but increased the expression of tight junction proteins in the ileum, thus markedly improving intestinal inflammatory damage and reducing the systematic inflammatory factors. Ileal genomics and protein validation suggested that voglibose attenuated inositol-requiring protein 1α-X-box binding protein 1-mediated endoplasmic reticulum stress (ERS). Together, these results showed that voglibose enhanced the secretion of GLP-1, which contributed to the glycemic control in KKAy mice at least in part by regulating intestinal inflammation and the expression of ERS factors.

## 1. Introduction

Type 2 diabetes mellitus (T2DM) is a multifactorial disease characterized by hyperglycemia, β-cell dysfunction and insulin resistance, which is associated with obesity, chronic inflammation and metabolic homeostasis [1]. With the development of the entero-insular axis in the pathogenesis and progress of T2DM, intestinal homeostasis and function play important roles in antidiabetic therapy [2].

The altered composition of intestinal microbiota and, consequently, different concentrations of bacterial metabolites, such as short-chain fatty acids (SCFAs) and lipopolysaccharides (LPS), may notably modulate glucolipid metabolism by affecting intestinal permeability and inflammation [3], which are commonly observed in individuals with T2DM [4]. SCFAs including acetate, propionate and butyrate play essential roles in the secretion of intestinal peptides, such as the glucagon-like peptide 1 (GLP-1) and peptide YY, resulting in increased insulin secretion and β-cell survival [5,6]. In addition, the transcellular transport of SCFAs from gut epithelial cells to immune cells can also regulate the gut inflammatory response associated with the filtration and differentiation of macrophages [7]. Studies in mice and humans have revealed the critical role of macrophages in metabolic diseases such as inflammatory bowel disease, obesity and diabetes [8]. In the healthy intestine, most macrophages maintain anti-inflammatory and resident subpopulations, whereas those with the pro-inflammatory phenotype have elevated expression of the nuclear factor kappa B (NF-κB) and tumor necrosis factor-alpha (TNF-α) when eating a high-fat diet (HFD) [9]. Colonic pro-inflammatory macrophages regulate glycemic control in mice fed an HFD as their numbers increase and alter glucose tolerance and peripheral insulin resistance [10,11]. Thus, the differentiation from pro-inflammatory macrophages toward anti-inflammatory subsets initiated by SCFAs could be a novel therapeutic method for T2DM.

During T2DM, dysbiosis of the intestine is not only limited to the immunologic process and the level of gut hormones but also results from endoplasmic reticulum stress (ERS)-related energy metabolism [12]. Recent studies have provided an understanding of the relationship between inflammation and ERS. In brief, ERS can be one cause of increasing inflammatory factors and/or the consequence of inflammation induced by chronic glucolipotoxicity [13]. The expression of several inflammatory factors such as NF-κB, TNF-α and interleukin 6 (IL-6) can be modulated by ERS and the consequent unfolded protein responses (UPR)-mediated signaling pathway [13]. In parallel with this result, a new study revealed that gut bacterial metabolites impact inflammatory bowel disease by regulating the ERS pathway [14]. Overall, the increasing inflammatory state and ERS caused by changing gut bacterial metabolites might destroy intestinal function and ultimately aggravate systemic inflammation and T2DM.

Voglibose, an anti-diabetic drug targeting α-glycosidase activity, mainly acts on the gastrointestinal tract to increase plasma GLP-1 content and inhibit dipeptidyl peptidase-4 activity [15]. A study in azoxymethane-induced colorectal preneoplastic lesions db/db mice showed that chronic treatment with voglibose tended to alleviate colorectal cancer by inhibiting insulin-like growth factor-mediated cell proliferation and intestinal inflammation [16], but its effects on T2DM associated with intestinal inflammatory damage are not entirely clear.

In this study, we evaluated the antidiabetic effects and underlying mechanisms of voglibose, especially on the regulation of fecal SCFAs, intestinal barrier integrity and inflammation and intestinal ERS in type 2 diabetic KKAy mice.

## 2. Results

### 2.1. Voglibose Ameliorates Glucose Metabolism in Diabetic KKAy Mice

**Figure 1:**
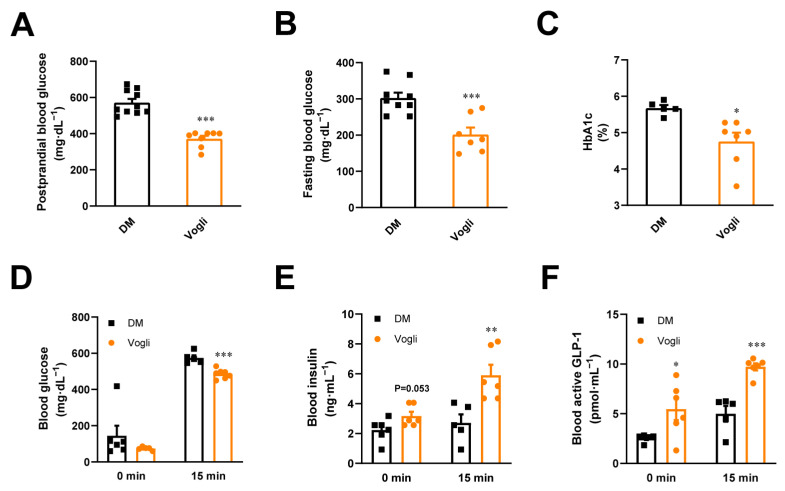
Voglibose ameliorates glucose metabolism in KKAy mice. (**A**) Fasting blood glucose. (**B**) Postprandial blood glucose. (**C**) HbA1c. (**D**) Blood glucose. (**E**) Blood insulin level at 0 and 15 min in the oral glucose-stimulated insulin secretion test. (**F**) Blood active GLP-1 level at 0 and 15 min in the oral glucose-stimulated GLP-1 secretion test. Data are expressed as the mean ± standard error of the mean (SEM) and individual data was shown in square (DM) and circle (Vogli), *n* = 5–10. * *p* < 0.05, ** *p* < 0.01, *** *p* < 0.001 vs. DM. DM, diabetic model group; Vogli, voglibose-treated group.

After treatment with voglibose for 28 days, the levels of fasting blood glucose (*p* < 0.001) and postprandial blood glucose (*p* < 0.001) were significantly decreased in the voglibose-treated group compared to the DM group (Figure 1A,B). Furthermore, the glycated hemoglobin A1c (HbA1c) (*p* < 0.05) level of KKAy mice in the voglibose group was lower than that in the DM group (Figure 1C), indicating that voglibose had long-term effects on glycemic control resulting from lowering the levels of blood glucose.

Compared to the DM group, voglibose also significantly reduced the blood glucose level at 15 min in the oral glucose tolerance test after a 6-week treatment (*p* < 0.001; Figure 1D) in diabetic KKAy mice. We further monitored the insulin and active GLP-1 levels to estimate the effects of voglibose on the secretion of insulin and GLP-1 in response to glucose. As shown in Figure 1E, voglibose tended to increase fasting blood insulin while significantly elevating blood insulin at 15 min after oral glucose loading compared to the DM group. As shown in Figure 1F, the serum level of active GLP-1 was increased at 15 min after oral glucose loading compared to 0 min, and voglibose significantly enhanced GLP-1 secretion by 124% (*p* < 0.05) at 0 min and by 94% (*p* < 0.001) at 15 min compared to the DM group. In addition, voglibose treatment notably decreased the body weight of KKAy mice at the end of the experiment (*p* < 0.001; Supplemental Figure S1).

### 2.2. Voglibose Modulates the Composition and Transposition of SCFAs

**Figure 2:**
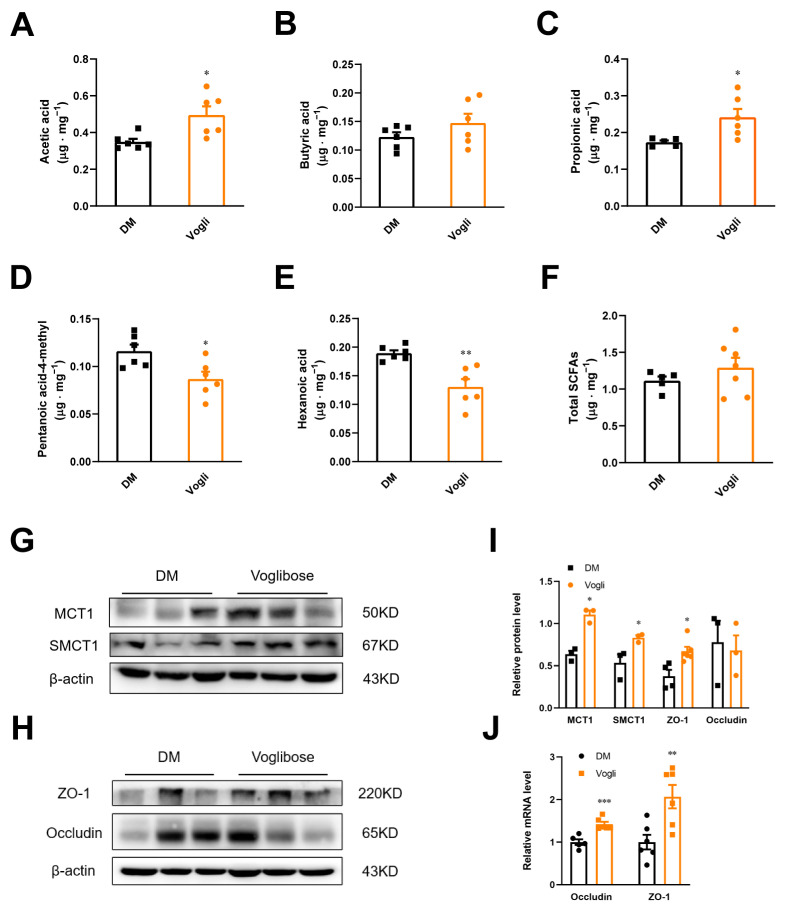
Voglibose alters the composition and transport of SCFAs in KKAy mice. Fecal SCFA concentra-tions including (**A**) acetic acid, (**B**) butyric acid, (**C**) propionic acid, (**D**) pentanoic acid-4-methyl, (**E**) hexanoic acid and (**F**) total SCFAs were quantified. Data are expressed as the mean ± SEM, *n* = 5–6. (**G**) MCT1, SMCT1, (**H**) ZO-1 and occludin protein abundance in the ileum tissue. (**I**) Quantification of proteins in (**G**,**H**). (**J**) Quantitative PCR analysis of occludin and ZO-1. Data are expressed as the mean ± SEM and individual data was shown in square (DM) and circle (Vogli), *n* = 3–6. * *p* < 0.05, ** *p* < 0.01, *** *p* < 0.001. DM, diabetic model group; Vogli, voglibose-treated group.

SCFAs are one of the metabolites generated by bacterial fermentation of dietary fiber in the gut. SCFAs also modulate the secretion of GLP-1. After observing the increased level of blood-active GLP-1 in KKAy mice, the concentrations of fecal SCFAs were consequently quantified as shown in Figure 2A–F. The concentrations of acetic acid (*p* < 0.05; Figure 2A) and propionic acid (*p* < 0.05; Figure 2C) were significantly increased in the voglibose-treated group, whereas isohexanoic (pentanoic acid-4-methyl) (*p* < 0.05) and hexanoic acids (*p* < 0.05; Figure 2D,E) were significantly decreased compared to the DM group. Voglibose tended to increase butyric acid and total SCFA levels compared to the DM group; although, the difference was not statistically significant (Figure 2B,F).

Several transport systems participate in the uptake of SCFAs including the monocarboxylate transporter 1 (MCT1) and sodium-coupled monocarboxylate transporter 1 (SMCT1). We assessed the ileal protein expression of MCT1 and SMCT1 in the two groups of KKAy mice to evaluate the impact of voglibose on the biological effects of SCFAs. Voglibose treatment notably increased the protein abundance of MCT1 and SMCT1 in the ileum compared to the DM group (Figure 2G,I). Given that SCFA levels are also associated with intestinal permeability, we detected the expression of zonula occludens-1 (ZO-1) and occludin (tight junction components). As shown in Figure 2H–J, compared to the DM group, voglibose markedly enhanced the protein (*p* < 0.05) and gene (*p* < 0.01) expression of ZO-1 and elevated the gene expression of occludin (*p* < 0.001), whereas the protein expression of occludin had no obvious variation.

### 2.3. Voglibose Alleviates Ileal Inflammation and Maintains Intestinal Integrity in Diabetic KKAy Mice

**Figure 3:**
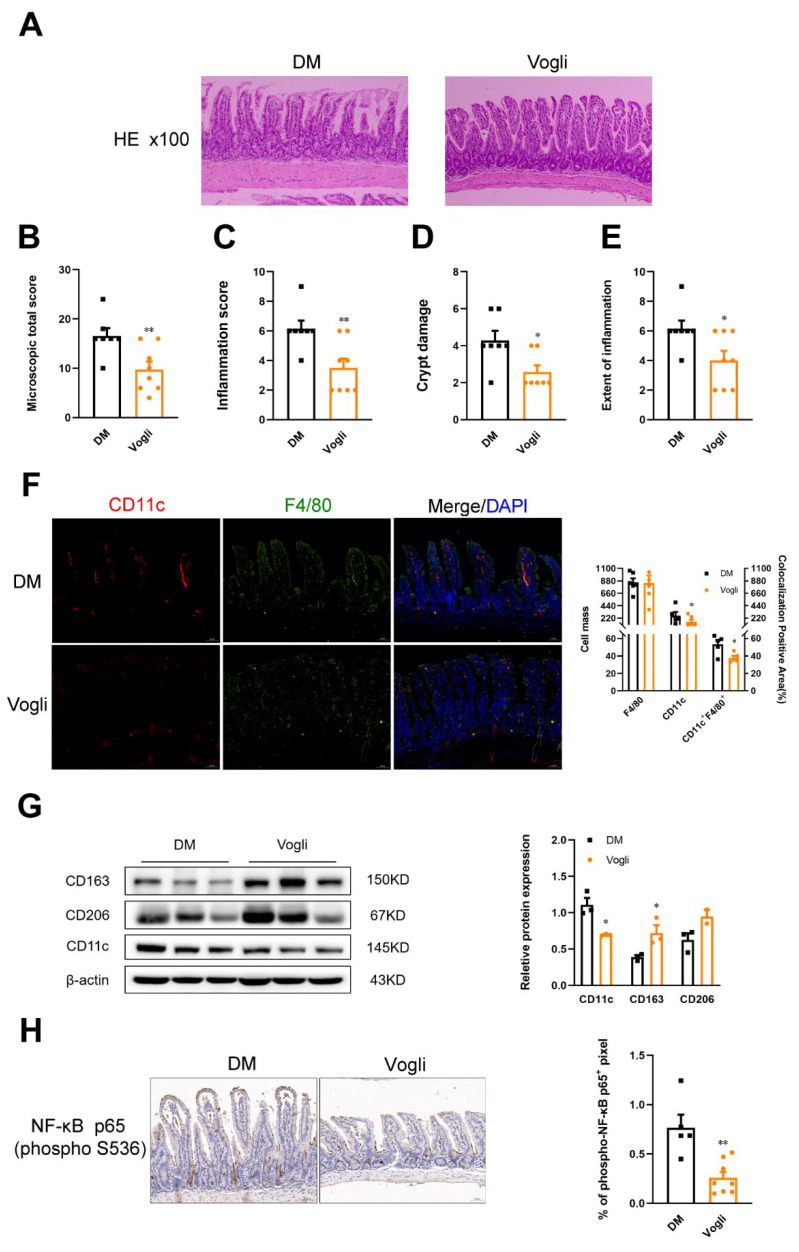
Voglibose alleviates ileal inflammatory injury and maintains intestinal integrity in KKAy mice. H&E staining of the ileal sections is shown (scale bar, 200 µm) (**A**), and histopathological assessment of the total score (**B**), inflammation score (**C**), crypt damages score (**D**) and extent of inflammation score (**E**) of the ileal sections were measured. (**F**) Immunofluorescence staining of CD11c (red), F4/80 (green) and DAPI (blue) of ileal tissue with the quantification of CD11c (CD11c^+^) or/and F4/80 (F4/80^+^) positive cells. (**G**) Protein abundance of CD163, CD206 and CD11c. (**H**) Immunohistochemical staining of p-NF-κB (p-S536) p65, and the quantification of p-NF-κB p65 positive area per field, *n* = 5–8. Data are expressed as the mean ± SEM and individual data was shown in square (DM) and circle (Vogli), * *p* < 0.05, ** *p* < 0.01 vs. DM. DM, diabetic model group; Vogli, voglibose-treated group.

The known effects of SCFAs in the intestine include enhancing the gut barrier function and exerting anti-inflammatory effects by acting on ileal epithelial cells, enteroendocrine cells and immune cells [11]. To evaluate the effects of voglibose on ileal function, the histological alteration of ileum tissue was evaluated using hematoxylin and eosin (H&E) staining. As shown in Figure 3A, the typical histoarchitecture loss was observed in the DM group, such as injury in the epithelium, fusion of the crypts, hypertrophy in the submucosal and mucosal layers and inflammatory cellular infiltrate. Several indexes were calculated based on the histological images (Figure 3B–E) to directly evaluate intestinal function. Treatment with voglibose inhibited damage to the ileum and significantly reduced the microscopic total score (*p* < 0.01), inflammation score (*p* < 0.01), crypt damage (*p* < 0.05) and extent of inflammation (*p* < 0.05) of ileal sections, indicating that voglibose ameliorated the progress of inflammation and protected the ileal integrity.

Considering the pivotal roles of macrophage infiltration in intestinal inflammation, the expression of specific factors of macrophages was detected using immunofluorescence and Western blotting. As shown in Figure 3F, compared to the DM group, fewer CD11c^+^ F4/80^+^ macrophages (*p* < 0.05) were observed in the voglibose-treated group, which is representative of the pro-inflammatory (M1) macrophage phenotype. In parallel to these results, the ileal protein expression of CD11c (*p* < 0.05) was lowered by voglibose, and the protein expression of CD163 (*p* < 0.05) and CD206, which are highly expressed in anti-inflammatory (M2) macrophages, were upregulated relative to the DM group (Figure 3G). We also assayed the expression of activated NF-κB p65 using immunohistochemical staining (Figure 3H). The positive area of phosphorylated NF-κB (p-NF-κB) p65 (*p* < 0.01) was decreased after long-term treatment with voglibose. Collectively, these results indicated that voglibose improved intestinal integrity and reduced inflammatory injury, which was associated with macrophage infiltration.

### 2.4. Voglibose Attenuates Endotoxin Content, Pro-Inflammatory Cytokines and Chemokine Levels in the Serum of Diabetic KKAy Mice

**Figure 4:**
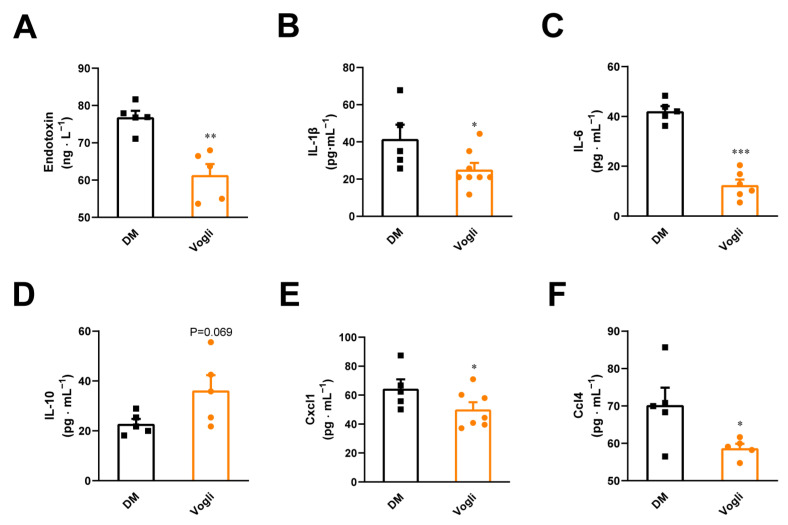
Voglibose improves inflammatory status in KKAy mice. (**A**) Endotoxin, (**B**) IL-1β, (**C**) IL-6, (**D**) IL-10, (**E**) Cxcl1, (**F**) Ccl4. Data are expressed as the mean ± SEM and individual data was shown in square (DM) and circle (Vogli), *n* = 5–8, * *p* < 0.05, ** *p* < 0.01, *** *p* < 0.001, vs. DM. DM, diabetic model group; Vogli, voglibose-treated group.

We detected the serum levels of endotoxin, cytokines and chemokines in KKAy mice, which are closely associated with the macrophage-dependent inflammatory response. Relative to the DM group, the serum content of endotoxin (*p* < 0.05; Figure 4A), which is also involved in HFD-induced diabetes, was reduced by voglibose. Additionally, multiple cytokines and chemokines such as IL-1β (*p* < 0.05; Figure 4B), IL-6 (*p* < 0.001; Figure 4C), C-X-C motif chemokine ligand 1 (CCXL1) (*p* < 0.05; Figure 4E) and C-C motif chemokine ligand 4 (CCL4) (*p* < 0.05; Figure 4F) were decreased in the voglibose-treated group. Moreover, we found an increasing trend in the serum levels of the anti-inflammatory factor IL-10 (Figure 4D) compared to the DM group. These results confirmed the effects of voglibose on attenuating inflammation.

### 2.5. Voglibose Mitigates the Ileal Expression of ERS-Related Factors in Diabetic KKAy Mice

**Figure 5:**
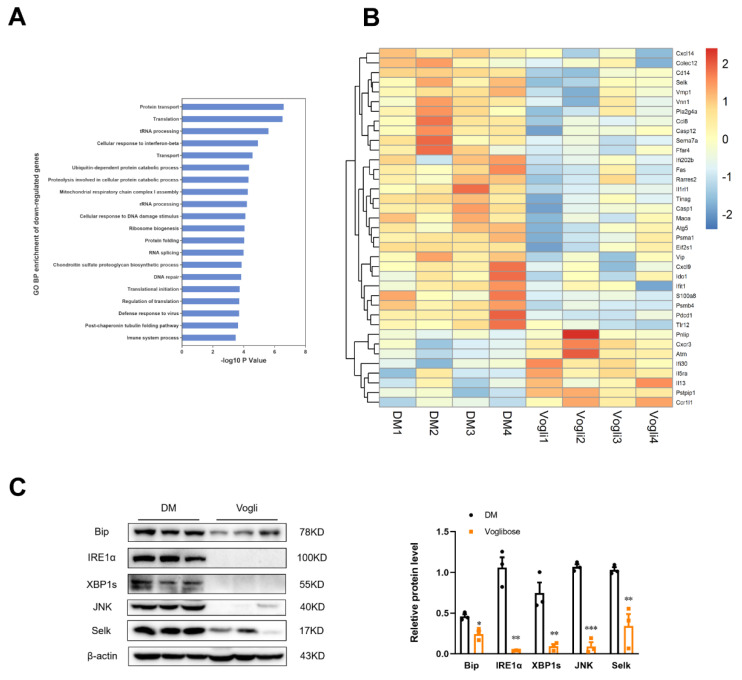
Voglibose decreases protein and gene expression of ERS-related factors. (**A**) Histogram of the enrichment of differential genes in GO biological processes. The results of the first 20 terms with the most significant enrichment are shown in the histogram (*p* < 0.05). (**B**) The heatmap for differentially expressed genes in the GeneChip microarray analysis. (**C**) Protein abundance of Bip, IRE1α, XBP1s, JNK and Selk. *n* = 3. Data are expressed as the mean ± SEM and individual data was shown in square (DM) and circle (Vogli), * *p* < 0.05, ** *p* < 0.01, *** *p* < 0.001 vs. DM. DM, diabetic model group; Vogli, voglibose-treated group.

The GeneChip microarray analysis was performed to further verify the molecular differentiation in the KKAy mice ileum between the voglibose-treated and DM groups, as well as reveal the function and underlying mechanism of voglibose in inflammatory injury and metabolic disorders in KKAy mice. A total of 22,207 detectable effective genes were obtained. Furthermore, 1528 genes were significantly differentially expressed genes (DEGs) (fold change > 1.2, *p* < 0.05) between the voglibose-treated group and the DM group, of which 603 were upregulated and 925 were downregulated (Supplemental Figure S3). Then Gene Ontology (GO) was conducted to annotate the function and determine the molecular characteristics of the DEGs. Figure 5A presents the top 20 enriched biological processes identified by the GO enrichment analysis based on the downregulated gene cluster, in which protein transport, transfer RNA processing, proteolysis involved in cellular protein catabolic process, protein folding and immune system process were involved. In addition, we also identified 37 DEGs that were mainly involved in the top 20 processes. These genes included UPR target genes such as selenoprotein K (Selk), caspase 12 (Casp12) and eukaryotic translation initiation factor 2 subunit alpha (Eif2s1); cytokine-cytokine receptor interaction-related genes such as Cxcl14, Ccl8, interleukin 1 receptor-like 1 (Il1rl1) and Il13; and Toll-like receptor (TLR) signaling pathway genes such as Cd14 and Tlr12 (Figure 5B). These results indicated that long-term treatment with voglibose in KKAy diabetic mice significantly altered the ileal gene expression, which was mainly associated with the UPR pathway and immunological regulation.

According to the above-mentioned results, we subsequently detected the protein expression of ERS-related factors. Voglibose significantly reduced (*p* < 0.05) the protein expression of binding immunoglobulin protein (Bip), an ER chaperone, which initiates the UPR by contacting the unfolded protein (Figure 5C). In addition, the expression of proteins that presented in the inositol-requiring enzyme 1-alpha (IRE1α)-mediated pathway were also clearly diminished by voglibose including the IRE1α (*p* < 0.01), spliced X-box-binding protein-1 (XBP1s; *p* <0.01) and c-Jun N-terminal kinase (JNK; *p* < 0.001). Except for the UPR, we also assessed the protein expression of SelK, one of the ER-resident selenoproteins that regulate ER-associated protein degradation and cell survival. The results showed that voglibose notably reduced (*p* < 0.01) its protein expression. These results in part indicated that voglibose potentially mitigated the ileal ERS state in diabetic KKAy mice.

## 3. Discussion

T2DM is the most common type of diabetes, and its prevalence has risen dramatically worldwide in recent years [17,18]. As a type of multifactorial metabolic disease, the occurrence and development of T2DM are usually accompanied by obesity, inflammatory injury and many diabetic complications [17]. Thus, the extension of potential targets of approved antidiabetic drugs and the discovery of multitarget therapies have become more essential.

Alpha-glucosidase inhibitors (AGIs) are oral antidiabetic drugs that mainly affect the intestine and inhibit the conversion of carbohydrates into monosaccharides to prevent the rapid increase of postprandial blood glucose in T2DM patients [16,19]. Several traditional Chinese medicines that potentially target α-glucosidase, such as Ramulus mori (Sangzhi) alkaloid (SZ-A), Berberine and Fucoidan, ameliorate glucose metabolism not only by preventing the intestinal digestion of carbohydrates but also by playing an important role in maintaining the intestinal homeostasis [16,20,21,22]. In addition, the inhibition of α-glucosidase induces the secretion of GLP-1 by moving unprocessed nutrients to the terminal part of the gut, where more L-cells are located and there is a prolonged duration of contact with nutrients [23]. Voglibose, one of the approved AGIs, acts on intestinal inflammation and affects the development of colorectal cancer in diabetic and colorectal pre-neoplastic db/db mice [16]. Collectively, we hypothesize that voglibose may control hyperglycemia by alleviating intestinal inflammatory injury, which is independent of its α-glucosidase inhibitory activity.

In this study, we first evaluated the pharmacological effect of voglibose on glucose metabolism in diabetic KKAy mice. We found that voglibose significantly reduced body weight and the fasting- and glucose-stimulated blood glucose and HbA1c while increasing the level of blood insulin and GLP-1 in response to oral glucose loading (Figure 1 and Supplemental Figure S1) in KKAy mice. Furthermore, no change was observed in fasting insulin or HOMA-IR between the voglibose group and the DM group (Figure 1 and Supplemental Figure S1). These findings indicate that long-term treatment with voglibose initiates better glycemic control, which is partly due to the increased GLP-1 level.

GLP-1 is an incretin hormone secreted by enteroendocrine L-cells with broad pharmacological effects on T2DM therapy [24]. Studies in mice and humans have shown that the interaction between active GLP-1 and GLP-1 receptors initiates numerous metabolic effects such as increased glucose-dependent stimulation of insulin, inhibition of appetite, delay of gastric emptying and decreased inflammation in the gut track [25]. The secretion of endogenous GLP-1 is strongly associated with intestinal metabolic homeostasis [25]. Consequently, the levels of SCFAs, one of the metabolites of intestinal bacterial fermentation, were investigated to reflect the differential microbiota metabolism between the voglibose-treated group and the DM group. As the results in Figure 2A–F show, treatment with voglibose significantly increased the concentrations of acetic and propionic acids, which contributed to basal and blood glucose-stimulated active GLP-1 in KKAy mice [5]. After identifying that the levels of isohexanoic and hexanoic acids were decreased, we then determined the expression of the translocators which are responsible for the transcellular transient of SCFAs, such as MCT1 and SMCT1, as shown in Figure 2G. Voglibose upregulated the ileal protein expression of MCT1 and SMCT1. Thus, we further confirmed that voglibose altered the intestinal metabolic state by regulating the concentration of SCFAs and their uptake between intestinal cells, which may result in increased GLP-1.

The intestinal barrier defense is initiated by the mucus layer, intestinal epithelial cells, tight junctions, immune cells and gut microbiota, whose pathological features and biological functions are closely associated with T2DM [26]. Epithelial tight junction proteins play essential roles in intestinal permeability, which allows the paracellular transport of some molecules and inhibits proteins, lipids and microbial-derived peptides [27]. In addition, a study of ApcMin/+ mice (a colorectal cancer mouse model) showed that 1% sodium butyrate treatment can enhance the tight junction complex and the associated adhesion molecules E-cadherin, which indicated that SCFAs potentially modulated the protein expression of the tight junction protein [28]. In accordance with the conclusion of this study, we found significant downregulation of the expression of ZO-1 and occludin produced by voglibose (Figure 2I). Subsequently, H&E staining was conducted to further evaluate the integrity of the intestinal barrier. As shown in Figure 3A, lower villus height, crypt depth and irregular crypt arrangement were observed in HFD-induced diabetic mice, which lessened with voglibose treatment [4]. Regarding the inflammatory state, several indexes of ileal H&E staining sections were calculated and proved to be decreased by voglibose including the inflammation score and extent of inflammation (Figure 3B–E). In correlation with our hypothesis, voglibose improved the integrity and permeability of the ileum in KKAy mice.

In addition to the effect of stimulating the release of gut peptides and expression of tight junction proteins, SCFAs, especially acetic and propionic acids, exert potent roles in inflammatory regulation by contacting with GPR43, namely, free the fatty acid receptor 2, which is highly expressed on immune cells (T cells, B cells, dendritic cells and macrophages) [29]. Macrophages are indispensable in preventing excessive intestinal immune response due to the ingestion and killing of pathogens. Therefore, we detected the proportion and expression of specific factors of macrophages via immunofluorescence and Western blotting. Voglibose decreased the total expression of CD11c in the ileal and the population of CD11c^+^ macrophages in F4/80^+^ macrophages (Figure 3F), which is representative of the pro-inflammatory (M1) macrophage phenotype. Although the population of anti-inflammatory (M2) macrophages has not been detected, the expression of M2 macrophage markers such as CD163 and CD206 has been observed to be upregulated (Figure 3G) by voglibose [30]. Considering the close relationship with the macrophage-mediated immune response, the NF-κB signaling pathway was also detected in the ileum [31]. The phosphorylation of p65 was significantly decreased by voglibose treatment (Figure 3H). Therefore, we determined that voglibose improved ileal inflammatory injury in KKAy mice by reducing monocyte recruitment and inhibiting inflammatory signaling mediated by NF-κB.

Damage to the intestinal barrier results in the leakage of gut microbiota-derived LPS and other toxins into the bloodstream, which triggers the secretion of pro-inflammatory cytokines and facilitates systematic inflammation [32,33]. Chronic low-grade inflammation is one of the complications of T2DM, which may in turn aggravate the progress of T2DM by leading to a lower mass of β-cells and insulin resistance [34,35]. In our study, we detected several inflammatory factors in both the blood and intestine tissue that evaluate the inflammatory state in and out of the intestine, respectively. As shown in Figure 4, voglibose treatment decreased the blood levels of endotoxin, pro-inflammatory cytokines and chemokines, including IL-1β, IL-6, Cxcl1 and Ccl4, and tended to elevate the level of anti-inflammatory factor IL-10 in KKAy mice. Voglibose treatment also significantly reduced the gene expression of IL-1β, whereas the gene expression of other related factors including IL-6, TNF-α, CD36 and CD86 were not obviously changed (Supplemental Figure S2). Collectively, these results further verified our hypothesis that voglibose initiates glycemic control by alleviating intestinal inflammatory injury and systematic inflammation.

Finally, the molecular characteristics of KKAy ileum were revealed through the microarray GeneChip. Analysis of DEGs showed that several pro-/anti-inflammatory genes participating in the cytokine–cytokine receptor interaction and TLR signaling pathway were decreased/increased, in accordance with the results of our in vivo study. Of note, the most downregulated abundant GO annotation of DEGs pointed to several biological processes such as protein transport and protein folding, indicating the potential effects of voglibose on the protein biosynthesis process. Consistently, differential gene analysis also revealed several genes in the UPR pathway such as Selk, Casp12 and Eif2s1. Given the complex signal network and dynamic environment in the intestinal tract, the demand for protein synthesis and the accumulation of unfolded proteins may ultimately cause ER stress [36]. The UPR, initiated by ER stress, operates in parallel using three mediators namely the IRE1α, protein kinase R-like endoplasmic reticulum kinase and activating transcription factor 6, in which the IRE1α and downstream XBP1 axis have been extensively studied and are known to be closely associated with the immune response [13,37]. We found a reduction in the protein expression of IRE1α-mediated factors, such as Bip, IRE1α, XBP1 and JNK, and verified the decreased protein expression of Selk in the ileum of the voglibose-treated group. However, the protein expression of other UPR genes was not confirmed, which might be due to the variation of expression between the protein and gene levels. Considering the associated underlying mechanism between ER stress, inflammation and diabetes, we hypothesize that the mitigative effect of voglibose on ileum ER stress is associated with its observed pharmacological effects on glycemic control and inflammation, but the specific mechanism should be further researched.

Notably, in this study, we investigated the anti-inflammatory effect of voglibose on the ileum because of its main pharmacodynamic characteristics and site. However, based on the known features of KKAy mice, such as obesity and T2DM, we recommend further investigations focusing on the pharmacological effect and mechanism of voglibose on inflammation in white adipose tissue and systemic insulin resistance, which may better explain its beneficial effects on T2DM and T2DM complications.

In conclusion, our results showed that voglibose is effective in GLP-1 secretion and glycemic control at least in part by improving intestinal inflammation and ERS. Our results also provided more evidence of the multiple effects and mechanisms of voglibose in T2DM treatment. 

## 4. Materials and Methods

### 4.1. Animal Experimental Design

Male KKAy mice (30 g) aged 14 weeks were purchased from Beijing Huafukang Bioscience Co., Ltd. (Beijing, China). Animals were housed in a temperature- and humidity-controlled environment at 23 ± 2 °C with a 12 h light/dark cycle. The KKAy mice were fed high-fat diets (45% of energy from fat; D12451; Research Diets, New Brunswick, NJ, USA) with free access to water. All animal experiments were performed in accordance with the guidelines for laboratory animals (GB14925-2001 and MOST 2006a) established by the People’s Republic of China and approved by the Institutional Animal Care and Use Committee of Institute of Materia Medica (Chinese Academy of Medical Sciences and Peking Union Medical College, Beijing, China).

After feeding a high-fat diet for 4 weeks, 16 KKAy mice were randomly divided into two groups (n = 8), either the diabetic model group (DM) or the voglibose-treated group (Vogli), according to the levels of blood glucose, total cholesterol, blood triglyceride body weight and percentage of blood glucose increase at 30 min after oral glucose loading. All mice were treated intragastrically with voglibose (1 mg/kg, lot number: 55120001, provided by SUZHOU CHUNG-HWA CHEMICAL&PHARMACEUTICAL INDUSTRIAL Co., Ltd., Suzhou, China) or an equivalent volume of water (0.05 mL/10 g body weight) once daily for 8 weeks.

### 4.2. Blood Glucose and Glycated Hemoglobin Measurements

The fasting blood glucose (FBG) and postprandial blood glucose (PBG) levels were dynamically monitored using the glucose-oxidase method (Biosino Bio-Technology and Science Inc., Beijing, China) during the experiment. After 42 days of treatment, the 0 h- and 4 h-fasted blood samples were collected from tail tips and glycated hemoglobin (HbA1c) levels were measured using commercial kits (A5911; Homa Biological, Beijing, China).

### 4.3. Oral Glucose-Stimulated Insulin and Glucagon-like Peptide 1 (GLP-1) Secretion Test

To evaluate the effect of voglibose on the secretion of insulin and GLP-1 response to glucose loading, oral glucose-stimulated insulin and GLP-1 secretion tests were conducted after 5 weeks of treatment. Before the experiment, all the mice were fasted overnight with water ad libitum. The D-glucose (2 g/kg) loading was given intragastrically followed by the collection of orbital blood samples at 0 min and 15 min. The DPP-4 inhibitor (DPP4-010, Millipore, Darmstadt, Germany) was added to the blood samples which were used to detect active GLP-1. The levels of serum insulin and active GLP-1 were assessed using ELISA kits (80-INSMSU-E10; 43-GP1HU-E01; ALPCO, Salem, MA, USA).

### 4.4. Histopathological Evaluation, Immunofluorescence, and Immunohistochemistry Assay of the Ileum

The ileum was taken from 10 cm of the intestine in front of the ileocecal valve and about 4 cm of ileum was isolated, fixed in 4% paraformaldehyde and embedded in paraffin to dissected into 5-μm-thick slides. Subsequently, hematoxylin and eosin (H&E) were performed on the sections of ileum for the analysis of inflammatory injury. Six random fields were selected from each section, and the histopathological evaluation of inflammatory and crypt damage was assessed as previously described. The ileum sections were also stained against F4/80 (ARG22476, Arigo Biolaboratories Corp, Taiwan, China) and CD11c (ARG59698, Arigo Biolaboratories Corp, Taiwan, China) for the immunofluorescence analysis and against NF-κB p65 (phosphor S536) (ab86299, Abcam, Cambridge, United Kingdom) for the immunohistochemistry analysis. Images were captured with a Mirax scanner (3DHISTECH, Budapest, Hungary), and the fluorescence intensities and positive area were analyzed with Image J software.

### 4.5. Fecal Short-Chain Fatty Acids Analysis

The fecal samples were collected from the ileum at the end of treatment and snap-frozen in liquid nitrogen. Fecal SCFAs were detected according to a previous report [38]. Briefly, fecal samples were crushed and extracted in methanol containing internal standards followed by ultrasonication and centrifuging at 4 °C. The SCFAs levels were determined using gas chromatography coupled to a mass spectrometer detector (GC–MS) (Agilent Technologies Inc., Santa Clara, CA, USA) and quantified using Masshunter quantitative software.

### 4.6. Serum Cytokines and Chemokines Assay

Blood samples were collected when the mice were sacrificed at the end of treatment followed by centrifuging at 4000 rpm to prepare the serum. The concentration of endotoxin was determined using an ELISA kit, and the concentrations of interleukin 1β (IL-1β), interleukin 10 (IL-10), interleukin 6 (IL-6), interleukin 12b (IL-12b), chemokine C-C-motif ligand 4 (Ccl4), chemokine C-C-motif ligand 5 (Ccl5) and CXC chemokine ligand 1 (CXCl1) in the serum were determined using Luminex liquid suspension chip detection, which was performed by Wayen Biotechnologies (Shanghai, China). The mouse 23-plex Multi-Analyte kit (Bio-Plex suspension Array System; Bio-rad, Hercules, CA, USA) was used following the manufacturer’s instructions.

### 4.7. Western Blotting and Quantitative Real-Time Polymerase Chain Reaction

The ileum tissues were homogenized in the RIPA lysis buffer with a protease and phosphatase inhibitor cocktail (Applygen Technologies Inc., Beijing, China), and the protein concentrations of the supernatant of lysate were measured and normalized using a BCA protein quantitation kit (Applygen Technologies Inc., Beijing, China). The protein samples were subsequently denatured and separated using sodium dodecyl sulfate polyacrylamide gel electrophoresis (SDS-PAGE). After transferring to a polyvinylidene difluoride (PVDF) membrane (Millipore, Darmstadt, Germany) and blocking with 5% nonfat milk for 2 h, the membranes were incubated with the primary antibodies overnight at 4 °C. The antibodies were obtained as follows: the anti-MCT1 (20139-1-AP), anti-SMCT1 (21433-1-AP) and anti-CD206 (60143-1-Ig) were from Proteintech Group Inc. (Beijing, China); and the anti-Zonula Occludens-1 (ZO-1, 61-7300, Invitrogen, Carlsbad, CA, USA), anti-occludin (33-1500, Invitrogen, Carlsbad, CA, USA), anti-selk (abs101948, absin, Shanghai, China), anti-CD163 (ab182422, Abcam, Cambridge, MA, USA), anti-XBP1 (ab220783, Abcam, Cambridge, MA, USA), anti-CD11c (97585), anti-Bip (3177), anit-Ire1α (3294) and anti-JNK (9252) were from CST (Danvers, MA, USA). The nonspecific combination was washed off followed by incubation with a horseradish peroxidase-conjugated secondary antibody (Applygen Technologies, Beijing, China). The protein signal was visualized using an enhanced chemiluminescence detection system (ChemiScope 2850; Clinx Science Instruments, Shanghai, China). The band densities were analyzed using ImageJ software, and the protein levels were normalized by β-actin.

Quantitative real-time PCR was performed as previously reported [39]. Briefly, total RNA was extracted from the ileum tissues using the TRizol reagent (15596018; Life Technology, Carlsbad, CA, USA) and reverse transcribed with TransScript^®^ first-strand cDNA Synthesis SuperMix (AT311; TransGen Biotec, Beijing, China). Quantitative real time-PCR was performed using the TransStart^®^ Tip Green qPCR SuperMix (TRANSGEN BIOTECH, Beijing, China) on a LightCycler^®^ 96 PCR System (Roche, Basel, Switzerland). The primers (Invitrogen, Beijing, China) used for gene amplification are listed in Table 1.

### 4.8. GeneChip Micoarray Analysis

All procedures of the GeneChip arrays and the data collection were conducted at the Beijing Cnkingbio Biotechnology Corporation. In brief, total RNA was extracted from the ileum tissues using the Trizol reagent (Life Technologies, Carlsbad, CA, USA) and purified with an RNeasy mini kit (Qiagen, Valencia, CA, USA). Biotinylated cDNAs were prepared according to the standard Affymetrix protocol from 250 ng total RNA using an Ambion^®^ WT Expression Kit. Following labeling, fragmented cDNA was hybridized for 16 h at 45 °C on a Clariom^TM^ S Assay (human, Affymetrix, Carlsbad, CA, USA). GeneChips were washed and stained in the Affymetrix Fluidics Station 450. All arrays were scanned using the Affymetrix^®^ GeneChip Command Console (AGCC) which was installed in the GeneChip^®^ Scanner 3000 7G. The row data(.cel) were normalized using the software TAC (Transcriptome Analysis Console; Vension:4.0.1) with the Robust Multichip Analysis (RMA) algorithm using the Affymetrix default analysis settings and global scaling as the normalization method.

For the microarrays, we used the R package limma (vension:3.36.5) to filter the differentially expressed genes (DEGs). The threshold for up- and down-regulated genes was set at a fold change > 2.0 and a *p*-value < 0.05. Hierarchical clustering was performed based on differentially expressed mRNAs using the R package pheatmap (vension:1.0.12). The GO analysis and pathway analysis were performed to analyze the primary function and significant pathway of the differential expression of mRNAs. Statistical analyses were performed using the two side Fisher’s exact test, and Benjamini–Hochberg was used for the multiple tests correction (FDR was used to adjust the *p*-values for multiple comparisons). The threshold set for significantly changed GOs was performed, and *p* <0.01 was considered as the significance threshold.

### 4.9. Statistical Analysis

All data are presented as the mean ± SEM. Statistical analysis was conducted using GraphPad Prism 8.0. *p*-values < 0.05 were considered to have statistical significance.

## Figures and Tables

**Table 1:** Primer sequences for qRT-PCR.

Gene	Primer sequence (5′—3′)
Occludin	Forward: ATGTCCGGCCGATGCTCTC Reverse: TTTGGCTGCTCTTGGGTCTGTAT
ZO-1	Forward: ACCCGAAACTGATGCTGTGGATAG Reverse: AAATGGCCGGGCAGAACTTGTGTA
β-actin	Forward: ACTCTTCCAGCCTTCCTTC Reverse: ATCTCCTTCTGCATCCTGTC

## Data Availability

The datasets presented in this study can be found in online repositories accessed on 15 October 2022. The names of the repository/repositories and accession number(s) can be found below: NCBI SRA; BioProject ID: GSE215823.

## Supplementary Materials

The following supporting information can be downloaded at: https://www.mdpi.com/article/10.3390/ijms232415938/s1, Supplemental Figure S1: Body weight and HOMA-IR of KKAy mice. Supplemental Figure S2: Gene expression in the ileum of KKAy mice. Supplemental Figure S3: Basic analysis of GeneChip microarray between the voglibose-treated group and the DM group.
