# Supplementary material for: Voglibose Regulates the Secretion of GLP-1 Accompanied by Amelioration of Ileal Inflammatory Damage and Endoplasmic Reticulum Stress in Diabetic KKAy Mice
Source: Int J Mol Sci. 2022 Dec 14;23(24):15938. doi: 10.3390/ijms232415938 (PMC9786790; doi:10.3390/ijms232415938)
Supplement: Supplementary file 1 [file ijms-23-15938-s001.zip › ijms-1998154-supplementary.pdf]

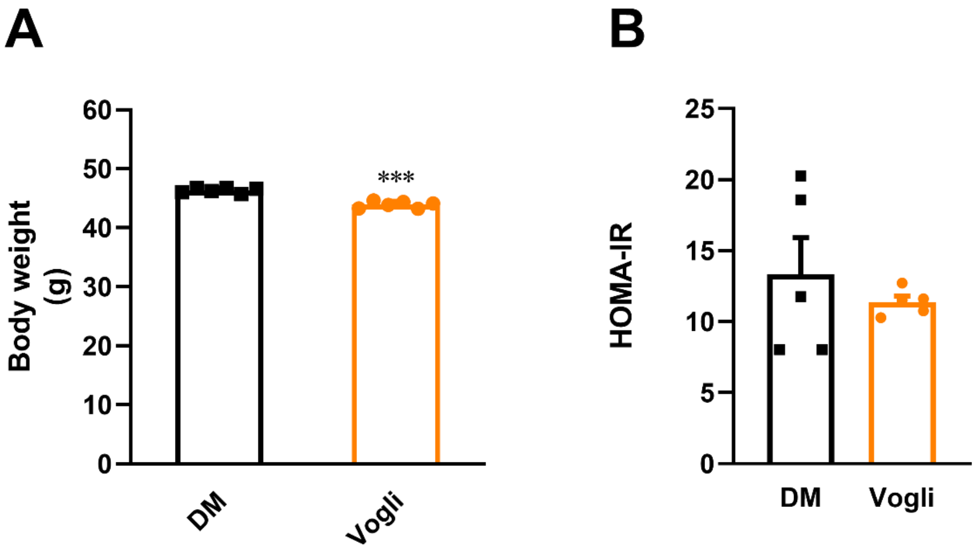

Figure S1. Body weight and HOMA-IR of KKAy mice. (A) Body weight (B) HOMA-IR. Data are expressed as the mean ± standard error of the mean (SEM), n = 5-6. ***$p < 0.001$ vs. DM. DM, diabetic model group; Vogli, voglibose-treated group.

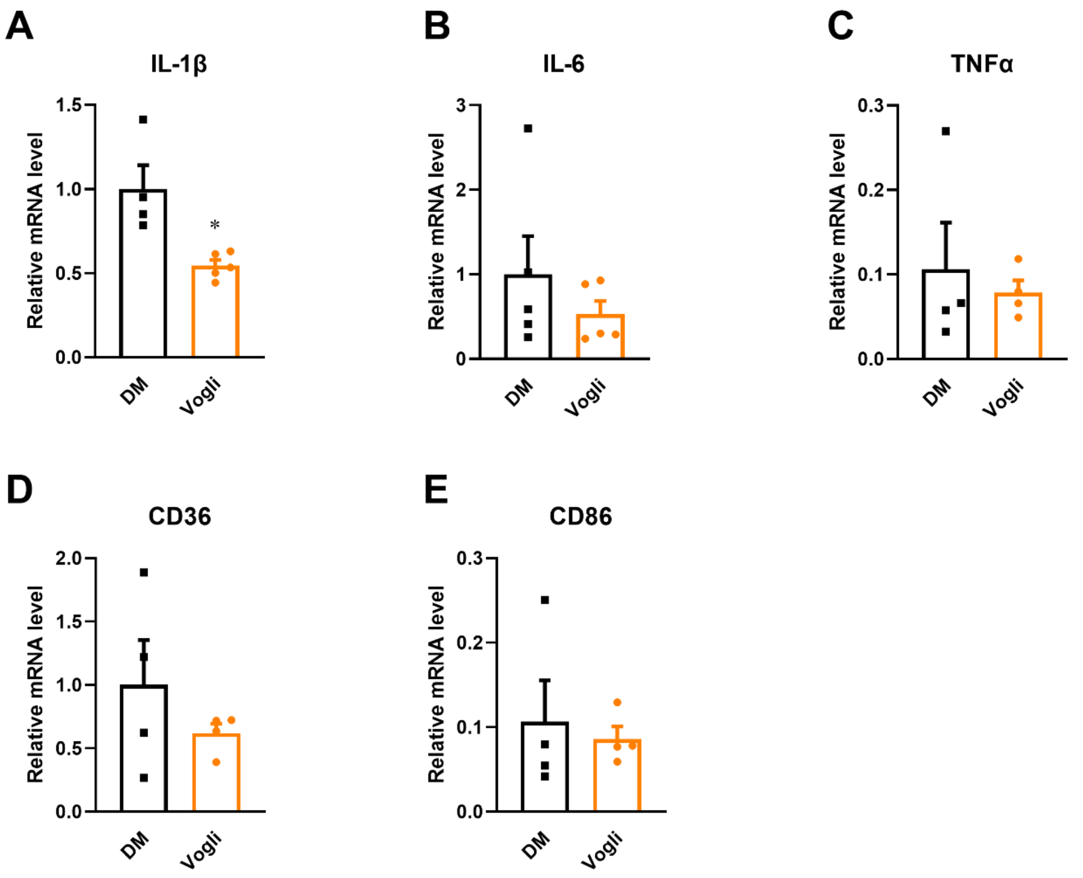

Figure S2. Gene expression in ileum of KKAy mice. (A) IL-1β, (B) IL-6, (C) TNFα, (D) CD36 and (E) CD86. Data are expressed as the mean ± standard error of the mean (SEM), n = 4-5. *$p < 0.05$ vs. DM. DM, diabetic model group; Vogli, voglibose-treated group.

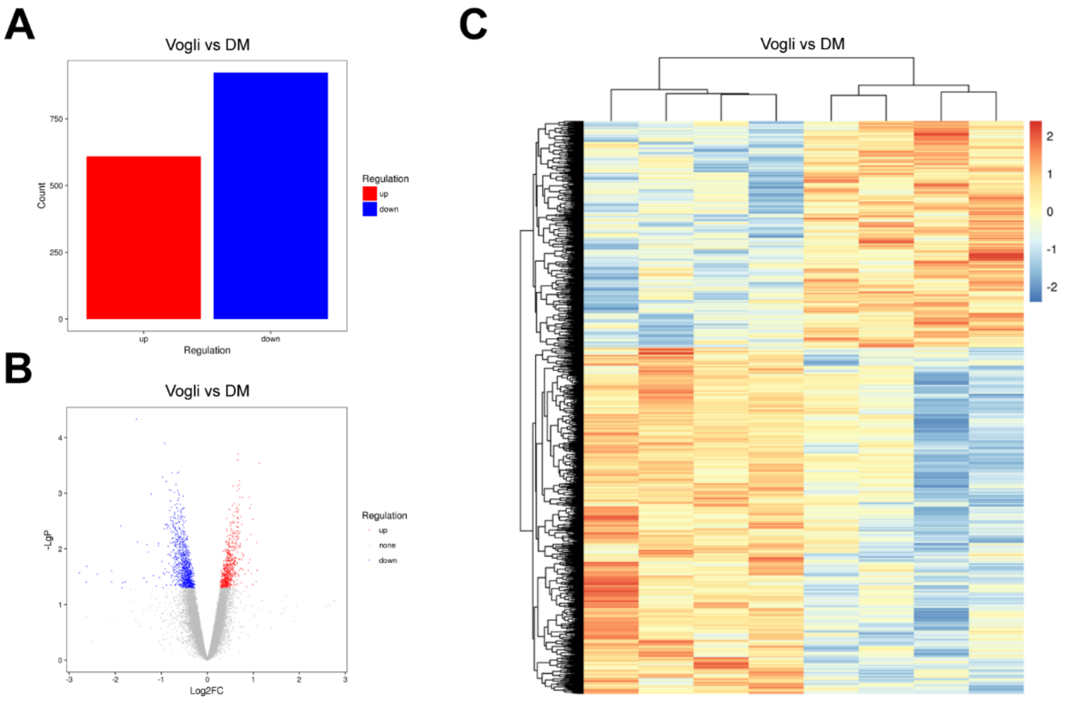

Figure S3. Basic analysis of genechip microarray between the Voglibose-treated group and the DM group. Histogram (A) and volcano plot (B) of up-regulated and down-regulated differentially expressed genes (DEGs) in voglibose-treated group compared with DM group. (C) Heatmap of total DEGs in voglibose-treated group compared with DM group. DM, diabetic model group; Vogli, voglibose-treated group.
